# Lithium as a disease-modifying therapy for Parkinson’s disease: mechanisms, preclinical evidence, and clinical prospects

**DOI:** 10.1016/j.prdoa.2026.100440

**Published:** 2026-04-09

**Authors:** Jackson T. Boonstra

**Affiliations:** Faculty of Behavioural and Movement Sciences, Vrije Universiteit Amsterdam, the Netherlands; Department of Neurology, Amsterdam University Medical Centers, the Netherlands

## Abstract

Parkinson’s disease (PD), the second most common neurodegenerative disorder, involves progressive dopaminergic neuron loss and α-synuclein aggregation. Current treatments, such as levodopa and dopamine agonists, offer symptomatic relief but do not slow neurodegeneration, underscoring the need for disease-modifying therapies. Lithium, a long-used mood stabilizer, shows neuroprotective potential via multiple mechanisms: inhibition of glycogen synthase kinase-3β (GSK-3β), enhancement of autophagy, suppression of neuroinflammation, and reduction of oxidative stress. Evidence from in vitro, animal, and early clinical studies suggests lithium can preserve dopaminergic neurons, improve motor function, and reduce neurodegenerative biomarkers, including serum neurofilament light chain. Preclinical models indicate that low-dose lithium (0.2–0.6 mM serum) improves striatal dopamine integrity, limits microglial activation, and decreases α-synuclein pathology, though study designs show substantial heterogeneity. Preliminary human trials with microdose lithium aspartate hint at slowed PD progression, though robust phase 3 data are lacking. Key challenges include optimizing formulations (*e.g.,* lithium orotate vs. carbonate), minimizing renal and thyroid risks, and identifying responsive patient subgroups. Comparative evaluation with other experimental therapies, such as glucagon-like peptide-1 agonists and immunotherapies, highlights lithium’s appeal as a cost-effective, repurposed candidate. This review synthesizes molecular and translational evidence, emphasizes research gaps such as long-term safety and standardized dosing, and aims to guide future clinical trial design and therapeutic strategies for PD.

## 1 Introduction

Parkinson's disease (PD) represents the second most prevalent neurodegenerative disorder globally after Alzheimer's disease, affecting approximately 1 million individuals in the United States and over 6 million people worldwide [1]. By 2050, demographic aging projections suggest this figure could reach 12 million, underscoring an impending public health crisis [1]. The disorder inflicts profound financial burdens, with U.S. healthcare costs exceeding $52 billion annually, alongside immeasurable emotional and caregiver strains [2]. Pathologically, PD is characterized by the progressive loss of dopaminergic neurons in the substantia nigra pars compacta. This loss is accompanied by intraneuronal α-synuclein aggregates, which form Lewy bodies, and a subsequent dysregulation of basal ganglia circuitry [3]. Clinically, PD manifests through cardinal motor symptoms, such as bradykinesia, resting tremor, rigidity, and postural instability. It also presents with debilitating non-motor features, including hyposmia, sleep disturbances, cognitive decline, and psychiatric symptoms [4]. While dopamine replacement therapies (*e.g.*, L-Dopa) provide symptomatic relief, their long-term use frequently triggers disabling motor fluctuations and dyskinesias [5]. Critically, no currently approved therapy halts or modifies the underlying neurodegenerative cascade, creating an urgent need for disease-modifying strategies [6].

Lithium, a monovalent cation approved for bipolar disorder treatment in 1970, has emerged as a compelling repurposing candidate for PD due to its pleiotropic neuroprotective properties (*i.e.,* lithium’s ability to target multiple disease pathways simultaneously) [7]. Beyond its mood-stabilizing effects, preclinical evidence demonstrates that lithium modulates multiple pathways implicated in PD pathogenesis through several key mechanisms. Lithium directly inhibits glycogen synthase kinase-3β (GSK-3β) at therapeutic concentrations (1–2 mM), reducing tau and α-synuclein hyperphosphorylation, key drivers of proteotoxicity in PD [8]. By disrupting GSK-3β-mediated inhibition of autophagy, lithium promotes clearance of α-synuclein aggregates, mitigating neuronal toxicity [9]. The compound exhibits anti-apoptotic effects by upregulating Bcl-2 expression while downregulating Bax, shifting the balance toward neuronal survival and preventing dopaminergic cell death in toxin models such as MPTP and 6-OHDA treatments [10]. Chronic low-dose lithium administration, maintaining serum levels between 0.2–0.6 mmol/L, attenuates microglial activation and pro-inflammatory cytokine release including TNF-α and IL-6, thereby reducing neuroinflammatory damage [11]. Additionally, lithium activates the NRF-2 pathway, bolstering antioxidant defenses against reactive oxygen species implicated in dopaminergic degeneration [12].

Despite robust preclinical evidence, translating lithium's benefits to PD patients presents challenges. Early clinical studies reveal a dose-dependent dichotomy. Low-dose lithium (serum levels < 0.6 mmol/L) demonstrates neuroprotection in rodent models and biomarker improvements in pilot human trials. However, higher therapeutic doses (0.8–1.2 mmol/L) carry risks of neurotoxicity, including irreversible cerebellar damage and drug-induced parkinsonism [13]. Notably, a retrospective cohort study of 1,749 elderly lithium users revealed an 87% increased incidence of dopaminergic drug prescriptions (HR: 1.87; 95% CI: 1.06–3.30), suggesting potential misdiagnosis of lithium-induced tremor as PD, a concerning prescribing cascade [14]. This risk appears heightened in older adults due to age-related declines in renal clearance, leading to elevated brain lithium levels despite “therapeutic” serum concentrations [15]. Nevertheless, recent innovations aim to circumvent these limitations through low-dose formulations (*e.g.*, lithium aspartate 20–45 mg/day) and novel delivery systems (*e.g.*, orotate salts) that enhance blood–brain barrier penetration while minimizing toxicity [16].

Emerging clinical evidence supports lithium's disease-modifying potential. A 2025 biomarker study by [13] demonstrated that PD patients achieving serum lithium levels of 0.21–0.56 mmol/L exhibited a median 12.8% reduction in serum neurofilament light chain (NfL), a sensitive marker of axonal degeneration (p = 0.0001 vs. low-dose group) [13]. Concurrently, low-dose lithium adjunctive therapy reduced L-Dopa–induced dyskinesias by 40–50% in MPTP-lesioned mice via calpain-1 inhibition, preserving striatal tyrosine hydroxylase expression [17]. These findings catalyzed the International Linked Clinical Trials (iLCT) programme (a collaborative initiative between Cure Parkinson's and the Van Andel Institute) to prioritize a phase 1b trial (NCT06099886) evaluating lithium aspartate in 35 early-stage PD patients [18].

This essay seeks to integrate current research on lithium’s potential role as a disease-modifying treatment for PD. Lithium's multimodal mechanisms are examined through the lens of PD pathophysiology, then critically evaluate its preclinical and clinical evidence, and finally, safety considerations and pharmacovigilance insights during ongoing clinical trials.

## 2 Literature search strategy

To ensure a comprehensive and contemporary review of the literature, searches were conducted across major biomedical databases, including PubMed, Scopus, and Web of Science. The search strategy was designed to capture all relevant publications addressing the mechanistic, preclinical, and clinical evidence for lithium as a potential disease-modifying agent in PD. The core search terms were grouped into three primary concepts:

1. Disease: “Parkinson's Disease” OR Parkinson* OR parkinsonism

2. Intervention: Lithium OR Li OR “mood stabilizer” OR repurposing

3. Outcome/Mechanism: neuroprotection OR “disease-modifying” OR GSK-3β OR autophagy OR neuroinflammation OR alpha-synuclein OR “clinical trial” OR preclinical

These terms were combined using Boolean operators (AND and OR) to maximize sensitivity and specificity. The search was performed for all available literature up to the cutoff date of August 2025. Inclusion criteria focused on primary research articles, systematic reviews, and *meta*-analyses that discussed lithium's effects in PD models (in vitro, animal) or human clinical trials. Articles were excluded if they focused solely on lithium's psychiatric applications without a direct link to PD pathogenesis. A “snowballing” technique was further employed to enhance the search's comprehensiveness; reference lists of all identified key articles, systematic reviews, and relevant narrative reviews were manually cross-referenced to identify additional pertinent studies that may have been missed by the initial database search.

## 3 Preclinical mechanisms and evidence

### 3.1 Lithium’s multimodal mechanisms through the lens of PD pathophysiology

The pathogenesis of Parkinson’s disease (PD) is multifactorial, involving α-synuclein misfolding and aggregation, mitochondrial dysfunction, oxidative stress, chronic neuroinflammation, and dysregulated kinase signaling [3], [6]. Lithium’s appeal as a repurposed therapeutic candidate lies in its capacity to simultaneously target several of these converging pathological mechanisms.

#### 3.1.1 Inhibition of GSK-3β and modulation of protein aggregation

Glycogen synthase kinase-3β (GSK-3β) is a serine/threonine kinase playing a pivotal role in PD pathogenesis by promoting hyperphosphorylation of tau and α-synuclein proteins, accelerating their fibrillization and Lewy body formations [8], [19]; this dysregulated phosphorylation contributes to neuronal dysfunction and dopaminergic neuron death. Lithium has shown to directly inhibit GSK-3β activity by competing with magnesium at ATP-binding sites, effectively reducing pathological phosphorylation and enhancing proteostasis [20]. More recent preclinical studies demonstrate pharmacological inhibition of GSK-3β, using small molecules or genetic approaches, preserves dopaminergic neurons and decreases α-synuclein aggregation in established PD models [21], [22], [23].

#### 3.1.2 Enhancement of autophagic clearance

Defective autophagy plays a critical role in PD pathogenesis by contributing to accumulation of toxic protein aggregates, particularly α-synuclein, as well as damaged mitochondria that exacerbate neuronal dysfunction and death [24], [25]. Lithium indirectly enhances autophagy through inhibition of inositol monophosphatase, which reduces intracellular inositol levels and triggers mTOR-independent autophagy activation, promoting the clearance of misfolded proteins and dysfunctional organelles via lysosomal degradation [9], [26]. This autophagy induction is neuroprotective, as demonstrated by increased autophagic flux following chronic lithium administration in α-synuclein–overexpressing cell models and MPTP-lesioned mice, resulting in improved motor outcomes and reduced neurotoxicity [24], [26], [27]. Recent studies have further elucidated lithium’s potential in enhancing neuronal regeneration and modulating related neuroinflammatory processes through autophagy pathways, highlighting its therapeutic promise for PD and related neurodegenerative disorders [28], [29].

#### 3.1.3 Anti-inflammatory effects via microglial modulation

Sustained microglial activation in PD exacerbates neurodegeneration by releasing pro-inflammatory cytokines such as TNF-α, IL-1β, and IL-6, perpetuating neuronal damage and disease progression [[30], [31], [32]]. Lithium attenuates microglial activation and suppresses pro-inflammatory cytokine transcription through inhibition of NF-κB signaling pathways and GSK-3β–dependent mechanisms, providing neuroprotective effects in PD models [11], [30]. In toxin-induced PD animal models, low-dose lithium administration significantly reduces striatal microgliosis and astrocytic reactivity, correlating with preservation of nigrostriatal dopaminergic neurons and improved motor outcomes [33].

#### 3.1.4 Mitigation of oxidative stress and mitochondrial dysfunction

Reactive oxygen species (ROS) generated from mitochondrial complex I impairment are central to PD-related neuronal loss [34]. Lithium activates the NRF2–ARE antioxidant response pathway, enhancing transcription of cytoprotective genes including heme oxygenase-1 and superoxide dismutase, thereby mitigating oxidative stress [14], [35], [36]. In models of neurotoxicity induced by rotenone and reserpine, lithium treatment reduces lipid peroxidation, DNA damage, and ROS accumulation, while improving mitochondrial membrane potential and ATP production [22], [33].

#### 3.1.5 Regulation of synaptic and glutamatergic toxicity

Excitotoxic glutamatergic signaling contributes to calcium overload and selective dopaminergic neuron death in PD [37]. In experimental systems, lithium has been shown to blunt this pathway in two complementary ways: reducing NMDA receptor–mediated Ca^2+^ entry into neurons (thereby limiting immediate calcium overload that drives mitochondrial/ER stress and downstream protease activation), and with longer treatment, it enhances glutamate clearance by increasing presynaptic/glial glutamate uptake capacity (reducing ambient/extracellular glutamate that triggers excitotoxic receptor activation). These actions (observed in cellular and animal studies and summarized in recent reviews) translate into less activation of calcium-dependent proteases (*e.g.,* calpains) and caspase cascades implicated in PD-related neurodegeneration, consistent with a net neuroprotective effect in preclinical models [38], see also [40] review on calpain-mediated neurodegeneration.

#### 3.1.6 Epigenetic modulation of α-synuclein expression

Beyond its well-characterized post-translational effects, lithium modulates α-synuclein (SNCA) gene expression through epigenetic mechanisms, particularly via DNA methylation pathways. In chronic MPTP mouse models of PD, lithium treatment has been shown to increase methylation of the SNCA promoter region (most notably within intron 1) resulting in decreased α-synuclein mRNA and protein levels, that correlates with reduced dopaminergic neuron loss and improved motor function [27], [41]. This epigenetic regulation is further supported by evidence that lithium influences microRNA-mediated control of DNA methyltransferases, such as miR-148a’s inhibition of DNMT1, thereby enhancing SNCA methylation [27], [42]. Lithium’s multi-modal action thus extends beyond α-synuclein suppression to include mitochondrial bioenergetic modulation, oxidative stress responses, and neuroinflammatory pathways, positioning it as a unique neuroprotective candidate capable of targeting multiple upstream and downstream pathological nodes in PD [24], [36], [43]. Nonetheless, the translation of these epigenetic and neuroprotective effects into clinical benefits for human PD remains to be rigorously validated through adequately powered randomized controlled trials [44].

#### 3.1.7 Methodological considerations in preclinical research

Although preclinical studies consistently report neuroprotective effects of lithium across toxin-induced and genetic PD models, closer examination reveals substantial heterogeneity that complicates translation. Supplementary Table 1 summarizes the main preclinical and clinical studies evaluating lithium’s neuroprotective mechanisms in PD. In the *meta*-analysis by [17], dosing regimens ranged from acute high-dose exposures (2–4 mEq/kg) to chronic low-dose administration approximating human serum levels of 0.2–0.6 mmol/L. Such variability precludes firm conclusions regarding optimal dose–response relationships or therapeutic windows. Timing of administration is equally inconsistent: paradigms initiating lithium before toxin exposure generally show stronger neuroprotection than those beginning after symptom onset [22], [27], raising the possibility that lithium’s efficacy may be greater in prevention than in restoration, an important consideration given that most PD patients present after motor symptoms emerge. However, not all studies demonstrate neuroprotection; notably, Yong et al. found that chronic lithium pretreatment failed to protect dopaminergic neurons in the rat 6-OHDA model despite successfully inhibiting GSK-3β activity [71].

Outcome measures also differ in both scope and sensitivity. While motor assessments such as the rotarod, cylinder test, and open-field paradigms are relatively standardized, neurochemical endpoints vary widely, including striatal dopamine quantification by HPLC, tyrosine hydroxylase immunoreactivity, and dopamine transporter binding assays [11], [33]. Each captures different aspects of nigrostriatal integrity, while the absence of harmonized protocols limits cross-study comparability.

#### 3.1.8 Mechanistic specificity and dose–target relationships

Lithium’s breadth of action, including GSK-3β inhibition, enhancement of autophagy, and anti-inflammatory effects, is frequently cited as a potential therapeutic advantage in both psychiatric and neurodegenerative contexts. These pleiotropic (producing more than one) effects could, in theory, provide broader clinical benefits by engaging multiple disease-relevant pathways simultaneously [12], [45]. However, each of these mechanisms is also targeted by other investigational agents, such as tideglusib, a selective and irreversible GSK-3β inhibitor, and rapamycin, a potent inducer of autophagy via mTOR inhibition [12], [46]. This raises an important unresolved question: *does lithium’s multi-target profile confer genuine synergistic benefit, or are its effects largely redundant compared to more selective agents acting on individual pathways?* Direct head-to-head comparative studies are lacking. Additionally, the relationship between serum lithium concentrations and central nervous system target engagement remains insufficiently defined. While therapeutic ranges for bipolar disorder typically fall between 0.8–1.2 mmol/L, preclinical neuroprotective effects, such as reduced glutamate-induced excitotoxicity and increased neuronal survival, are often observed at lower concentrations (0.2–0.6 mmol/L) [47], [48]. This suggests the existence of distinct dose–effect profiles for mood stabilization versus neuroprotection, underscoring a need for more targeted pharmacokinetic–pharmacodynamic studies in humans to optimize dosing for specific clinical outcomes.

## 4 Clinical translation and prospects

### 4.1 Clinical evidence: biomarkers versus clinical endpoints

Recent clinical studies of lithium in PD, including those by [13], [18], have demonstrated modulation of candidate biomarkers such as serum neurofilament light chain (NfL) and transcriptional upregulation of neuroprotective genes (*e.g.,* Nurr1, SOD1). However, these biomarkers are not yet validated surrogates for PD progression. While NfL is broadly correlated with neurodegeneration, its specificity for dopaminergic neuronal loss and motor decline in PD remains uncertain, and regulatory agencies such as the FDA and EMA have not accepted it as a qualified endpoint [49], [50]. Tolerability remains a limiting factor. In [18], 33% of participants in the medium-dose arm withdrew, a rate that risks introducing selection bias toward patients with better baseline renal function or side-effect tolerance. Such attrition challenges generalizability, particularly in older PD cohorts where comorbidities are common.

### 4.2 Formulation and delivery challenges

Interest in alternative lithium salts such as lithium orotate and lithium aspartate reflects a desire to increase CNS delivery while limiting systemic exposure. Lithium orotate has been proposed to be relatively more lipophilic and to remain partly non-dissociated in biological fluids, a property that could favor membrane/blood–brain-barrier transit and higher brain lithium concentrations in some animal experiments [51], [52]. By contrast, “*microdose*” regimens of lithium aspartate (clinical dose ranges under active investigation ∼ 20–45 mg/day of the salt in some trials and open-label dose-finding work) have been tested recently to try to engage putative neuroprotective targets with minimal peripheral accumulation (NCT04273932) [18]. Crucially, there are no published human pharmacokinetic studies that directly compare brain-to-plasma ratios across lithium formulations (*e.g.,* orotate, aspartate, carbonate); dose selection for alternative salts therefore still largely depends on preclinical models and extrapolation, which may not translate predictably between species and raises unresolved safety questions [52], [53].

### 4.3 Comparative context in the disease-modifying landscape

Lithium’s development and therapeutic role must be understood within a competitive and evolving landscape of neuroprotective and disease-modifying agents, which include GLP-1 receptor agonists, LRRK2 inhibitors, α-synuclein immunotherapies, and iron chelators. In particular, GLP-1 receptor agonists such as liraglutide and semaglutide have garnered attention for sharing several neuroprotective mechanisms with lithium, including modulation of oxidative stress, mitochondrial protection, and cognitive improvement, while generally offering a more favorable tolerability profile, especially in older adults [37], [54], [55]. Emerging evidence indicates liraglutide can reverse mania-like behavioral alterations and cognitive deficits in animal models that resemble bipolar disorder and enhances the mood-stabilizing and neuroprotective effects of lithium when used in combination [56]. Additionally, GLP-1 receptor agonists have been implicated in modulating dopamine release through central mechanisms lithium also influences, suggesting potential convergent pathways mediating clinical outcomes [57]. Despite overlaps, lithium’s mechanisms involving GSK-3β inhibition and complex intracellular signaling cascades remain unique and integral to its efficacy profile, particularly in bipolar disorder and neuroprotection [58], [59]. However, without direct head-to-head clinical trials comparing lithium to newer agents like GLP-1 receptor agonists, delineating lithium’s distinct therapeutic niche and potential advantages remains speculative. Continued research to clarify these relationships and therapeutic complementarities is critical for optimizing neuropsychiatric and neurodegenerative disorder treatments [60], [61], [62].

### 4.4 Clinical trial design and regulatory hurdles

Robust clinical trials aiming for disease modification in PD typically require extended follow-up durations of 18–24 months or more and large sample sizes ranging from 100 to 400 patients per treatment arm to reliably detect meaningful differences in motor progression. This essential need stems from relatively slow and variable progression rate of PD in affected individuals, while clinical and phenotypic heterogeneity of PD, including key subtypes such as tremor-dominant versus postural instability/gait difficulty phenotypes, further complicate trial designs; stratifying patients by subtype often becomes necessary to reduce variability, though it adds layers of complexity and cost to trials [60], [61], [62], [63], [64], [65], [66].

Enrichment strategies based on genetic markers like LRRK2 and GBA mutations, or based on biomarkers indicative of inflammation and autophagy dysfunction, hold promise for improving signal-to-noise ratio in trials by targeting more homogeneous biologic subgroups, however, these strategies require validated, reliable stratification tools and biomarkers, that are currently under active development, but have yet to achieve broad regulatory endorsement [60], [61], [65]. One specific example is a neurofilament light chain (NfL) protein biomarker, a candidate surrogate endpoint that has not yet been officially qualified by regulatory agencies as a surrogate marker for accelerated approval pathways in PD [65], [66].

Regarding lithium therapeutics, academic–industry partnerships and public or philanthropic funding are essential to advance high-quality, large-scale clinical testing. Without commercial exclusivity, these collaborations become pivotal in sustaining and accelerating research development [60]. Long-term safety and tolerability must be demonstrated for interventions intended for chronic use in PD, an important challenge compounded by heterogeneous disease courses and variability in patient responses [65], [67]. Ongoing clinical trials targeting LRRK2 mutations exemplify many of these challenges: LRRK2 mutations present in a relatively small subset of PD patients, necessitating careful patient identification, while trials are limited by a lack of validated progression markers and measures of target engagement. Recruitment strategies must optimize timing, usually focusing on patients recently diagnosed with LRRK2-associated PD who have stable symptomatic management but lack motor fluctuations, to balance trial feasibility and meaningful clinical endpoints [60], [65].

## 5 Safety considerations and pharmacovigilance

### 5.1 Safety signals and the lithium–parkinsonism paradox

The first detailed description of lithium-induced parkinsonism was provided by [72], who reported a 71-year-old woman with disabling bradykinesia, rigidity, and rest tremor that resolved almost completely within five weeks of lithium discontinuation, despite therapeutic serum levels. This landmark observation established the clinical plausibility of lithium as a cause of reversible parkinsonism, independent of overt toxicity. Subsequent case reports have expanded this picture, describing both typical parkinsonian–like motor symptoms in elderly bipolar patients and atypical presentations such as vocal cord paralysis resolving after dose adjustment [73] or mixed rest–action tremor with symmetric onset [74], [75]. Lithium intoxication has also been associated with acute parkinsonism alongside hyperparathyroidism and nephrogenic diabetes insipidus, particularly in older adults on chronic therapy [76].

Observational pharmacovigilance data add further complexity. [77] reported an increased hazard ratio (1.87) for subsequent dopaminergic medication use among chronic lithium users. This could reflect true lithium-induced parkinsonism, closer neurological surveillance in this group, or psychiatric comorbidities that independently elevate PD risk. However, Japanese pharmacovigilance data suggest lithium carbonate itself may not directly increase Parkinson-like events, with such signals potentially arising from co-treatment with agents like sodium valproate [14].

### 5.2 Long-term safety and pharmacovigilance registries

An enduring challenge with lithium is its narrow therapeutic index and risk of long-term renal, thyroid, and neurological side effects, particularly in older adults [15]. The potential for misdiagnosis of lithium-induced tremor as PD highlights a critical need for rigorous safety monitoring [14]. To address these concerns, establishment of long-term safety registries could work to track PD patients receiving low-dose lithium over an extended period (*e.g.*, 5–10 years), allowing for collection of high-quality data on renal function, thyroid hormone levels, and neurological outcomes. Such registries would be invaluable for assessing true long-term safety profiles of lithium in a PD-specific population. Additionally, they would provide a robust platform for pharmacovigilance, enabling detection of rare side effects and providing a clearer picture of risk–benefit ratio of chronic lithium use in PD. Data from these registries, combined with findings from rigorous phase 3 trials, would need to be kept safe, and may prove essential for informing clinical practice and gaining regulatory approval for lithium as a repurposed DMT for PD.

### 5.3 Neurotoxicity thresholds and conflicting safety evidence

Lithium’s safety profile is defined by a narrow therapeutic index and contradictory evidence regarding its neurotoxicity thresholds. Serum levels between 0.6–1.2 mEq/L are traditionally considered therapeutic, yet neurotoxicity, including parkinsonism and cerebellar dysfunction, has occurred at levels as low as 0.4–0.6 mmol/L, especially in elderly patients with impaired renal function [78], [79]. While lithium’s therapeutic index is approximately 2.8, reflecting limited margin between efficacy and toxicity, low-dose exposure (0.2–0.6 mmol/L) has been associated with neuroprotection and reduced neurodegenerative biomarkers [11], [13]. This paradox underlies a central safety controversy: whether lithium’s beneficial signaling effects of GSK-3β inhibition, autophagy enhancement, and anti-inflammatory modulation can be separated from its toxic mechanisms, such as mitochondrial dysfunction and demyelination [12], [80].

Lithium-induced parkinsonism may occur within the “*therapeutic*” window, independent of overt toxicity, with risk increasing with age, chronic exposure, and polypharmacy [75], [77] Conversely, pharmacovigilance data from Japan found no direct association between lithium carbonate and Parkinson-like events when controlling for sodium valproate co-treatment, underscoring population-specific variability and potential confounding [14]. The emerging syndrome of irreversible lithium-effectuated neurotoxicity (SILENT) further blurs dose-toxicity boundaries, with persistent cerebellar and cognitive sequelae reported after both acute intoxication and normotherapeutic use [81], [82]. Together, these findings emphasize that serum lithium concentration alone does not fully predict neurotoxic risk; individual pharmacokinetics, comorbidities, and co-administered agents critically shape outcomes. Ongoing debates therefore center on redefining “*safe*” exposure thresholds specific to neurodegenerative populations, where subtherapeutic dosing may maximize neuroprotective yield while minimizing cumulative neurotoxicity.

## 6 Recommendations for future research

In this section, key recommendations are outlined for future research essential for translating promising preclinical findings on lithium into a validated, safe, and effective disease-modifying therapy (DMT) for PD. Advancing lithium toward definitive clinical evaluation will require a multi-faceted approach, encompassing mechanistic validation, biomarker qualification, and innovative trial designs.

### 6.1 Mechanistic validation of central nervous system target engagement

A significant gap in current literature is the lack of direct evidence linking specific brain lithium concentrations to engagement of its purported therapeutic targets in the central nervous system. While preclinical studies have consistently demonstrated that lithium inhibits GSK-3β, enhances autophagy, and reduces inflammation, these effects are largely inferred from in vitro and animal models [9], [11], [20]. Future research should thus prioritize mechanistic validation studies. This can be achieved through techniques such as positron emission tomography (PET) imaging with tracers that bind to GSK-3β, allowing for direct, non-invasive measurement of target occupancy in the human brain. Additionally, longitudinal analysis of cerebrospinal fluid in patients receiving lithium could provide critical data on lithium concentrations and how they correlate with changes in key biomarkers such as phosphorylated α-synuclein and GSK-3β activity. Establishing a clear link between brain lithium levels and its intended molecular effects is crucial for defining optimal therapeutic window for neuroprotection, which appears to be distinct from higher concentrations required for mood stabilization (0.2–0.6 mmol/L vs. 0.8–1.2 mmol/L, respectively) [13], [55].

### 6.2 Biomarker qualification and validation for clinical endpoints

The development of disease-modifying therapies for PD is significantly hampered by the slow, variable nature of the disease and a lack of reliable surrogate endpoints for progression. While emerging human data from pilot trials suggest low-dose lithium can reduce serum neurofilament light chain (NfL), a marker of axonal degeneration, this biomarker has not yet been formally qualified by regulatory agencies as a surrogate for PD progression [13], [45], [46]. A key research priority should be the qualification of robust biomarkers that serve as reliable surrogates for clinical outcomes. This involves conducting large-scale, longitudinal studies to validate the utility of biomarkers like serum NfL, plasma phosphorylated α-synuclein, and CSF inflammatory markers. These studies should correlate changes in biomarker levels with long-term clinical endpoints, such as the Unified Parkinson's Disease Rating Scale (UPDRS) scores and time to motor complications. A qualified biomarker could dramatically accelerate clinical trials by reducing the required sample size and follow-up duration needed to detect a therapeutic effect. Given the high cost and complexity of large-scale trials, establishing a validated surrogate endpoint is essential for the feasibility and success of future lithium studies.

### 6.3 Precision medicine and patient stratification

The clinical heterogeneity of PD, including distinct subtypes (*e.g.,* tremor-dominant vs. postural instability/gait difficulty), complicates clinical trial design and can obscure a true therapeutic effect in an unselected population [68], [69], [70]. Furthermore, varied and multiplex effects of lithium suggest certain patient subgroups may be more likely to benefit from its mechanisms. Future clinical trials should move toward a precision medicine approach, stratifying patients based on biomarkers or genetic profiles that align with lithium's known mechanisms of action. For instance, trials could enrich for patients with high baseline levels of inflammatory markers (*e.g.,* TNF-α, IL-6), who may be more responsive to lithium's anti-inflammatory effects [11]. Similarly, patients with evidence of impaired autophagy or elevated α-synuclein aggregation could be ideal candidates for a lithium-based intervention [9]. Enrichment strategies based on genetic markers like LRRK2 and GBA mutations, or other biomarkers, are vital for improving the signal-to-noise ratio in trials, ensuring therapeutic effects are not diluted by a large group of non-responders. This targeted approach is already being applied in trials for other investigational agents and represents a future of more effective clinical research in PD [66], [67].

### 6.4 Comparative effectiveness and combination potential

The evolving landscape of disease-modifying therapies for PD includes several promising candidates, such as GLP-1 receptor agonists, LRRK2 inhibitors, and α-synuclein immunotherapies [60]. Given lithium shares some mechanisms with these agents (*e.g.,* modulation of oxidative stress and mitochondrial protection with GLP-1 agonists), it is critical to define its unique therapeutic niche. Research initiatives should support comparative effectiveness studies to directly evaluate lithium against other experimental agents. Head-to-head trials are necessary to determine if lithium's multi-target profile confers a genuine synergistic benefit or if its effects are redundant compared to more selective therapies. Furthermore, a deeper understanding of its mechanisms could inform design of combination therapy trials. For example, since lithium and GLP-1 receptor agonists appear to have complementary mechanisms, combining them could potentially yield greater neuroprotective effects than either monotherapy [62]. Delineating these therapeutic complementarities is essential for optimizing treatment strategies and for deciding where lithium fits best in the armamentarium of PD therapies.

## 7 Discussion

Lithium represents a compelling repurposing candidate for Parkinson’s disease due to its multimodal neuroprotective mechanisms and promising preclinical and early clinical evidence. Preclinical studies demonstrate low-dose lithium (serum levels 0.2–0.6 mmol/L) preserves dopaminergic neurons, reduces α-synuclein aggregation, and mitigates neuroinflammation and oxidative stress in toxin-induced (MPTP, rotenone) and genetic PD models [11], [22], [27], though protective effects are not universal across all neurotoxin paradigms [71]. Mechanistically, lithium inhibits glycogen synthase kinase-3β (GSK-3β), attenuating pathological phosphorylation of tau and α-synuclein [8], while enhancing autophagy to clear misfolded proteins and damaged mitochondria [9], [24]. Its anti-inflammatory effects, mediated through NF-κB suppression and microglial modulation [11], alongside NRF-2–driven antioxidant responses [12], further underscore its potential to target multiple pathways implicated in PD progression.

Early clinical trials support lithium’s translational promise. A pooled analysis by [13] reported a median 12.8% reduction in serum neurofilament light chain (NfL), a biomarker of axonal injury, in PD patients achieving serum lithium levels of 0.21–0.56 mmol/L. Pilot studies also suggest transcriptional upregulation of neuroprotective genes (*e.g.,* Nurr1, SOD1) and potential reductions in L-Dopa–induced dyskinesias via calpain-1 inhibition [17]. These findings have spurred ongoing clinical investigations, including a phase 1b trial of lithium aspartate in early PD [18].

However, significant challenges remain. Lithium’s narrow therapeutic index poses risks of renal, thyroid, and neurological toxicity, particularly in older adults with reduced renal clearance [15]. Lithium-induced parkinsonism (LIP), which can mimic idiopathic PD, has been documented even at therapeutic serum levels [14], [50], necessitating careful clinical monitoring. Optimal dosing and formulation (*e.g.,* carbonate vs. aspartate vs. orotate) require further study, as preliminary data suggest lithium aspartate may dissociate more readily, leading to higher central nervous system exposure and side effects at seemingly equivalent doses [18]. Biomarkers like NfL, while promising, lack regulatory validation as surrogate endpoints for PD progression [45], [46], complicating trial design.

Our synthesis advances beyond prior reviews by critically integrating the full spectrum of lithium's pleiotropic actions, particularly highlighting the emerging role of lithium in the epigenetic modulation of α-synuclein (SNCA) expression. Specifically, we elucidate how lithium influences DNA-methyltransferase activity and miRNA networks (*e.g.,* miR-21-5p, miR-146a-5p) that target CpG-rich promoter regions, thereby reducing SNCA transcription, an axis that complements the “*information-theory of aging*” framework. This transcriptional focus is then placed in direct comparison with other promising disease-modifying agents, such as Glucagon-like Peptide-1 (GLP-1) agonists. While both lithium and GLP-1 agonists converge on the critical GSK-3β hub to promote autophagic clearance, analysis reveals their distinct upstream signaling cues (*e.g.,* Akt activation by GLP-1) and primary modes of action. By comprehensively mapping converging and diverging mechanistic landscapes from the transcriptional-epigenetic axis to downstream clearance circuitry, this review provides a uniquely integrated framework for designing next-generation therapeutic strategies that could leverage the synergistic potential of these diverse disease-modifying candidates.

Future research should prioritize several key areas to advance lithium’s potential as a disease-modifying therapy for PD. First, mechanistic validation is needed to confirm target engagement (such as GSK-3β inhibition and autophagy induction) in human PD brains using advanced imaging techniques and CSF biomarker analysis. Second, a precision medicine approach should be employed, stratifying patients based on biomarkers (*e.g.,* inflammation or autophagy dysfunction) and/or genetic profiles (*e.g.,* LRRK2, GBA) to identify subgroups most likely to benefit from lithium therapy. Third, comparative effectiveness trials should evaluate lithium against other emerging disease-modifying candidates, such as GLP-1 agonists and LRRK2 inhibitors, to clarify its unique therapeutic niche. Finally, long-term safety must be rigorously assessed, potentially through dedicated registries, to monitor renal, thyroid, and neurological outcomes in PD-specific populations, ensuring benefits of lithium outweigh its risks in clinical practice. Addressing these priorities will be essential for determining whether lithium can fulfill its promise as a viable neuroprotective strategy for PD.

While lithium monotherapy may not suffice for broad PD treatment, its low cost and multifarious actions make it a viable adjunct or prophylactic option. A definitive phase 3 trial, incorporating biomarker-guided dosing and enriched patient cohorts, is essential to determine whether lithium can fulfill its potential as a disease-modifying therapy for PD. Until then, cautious optimism, tempered by rigorous safety monitoring, should guide investigational use.

**Funding source**

The author received no financial support for the research, authorship, and/or publication of this article.

## Declaration of competing interest

The author declares that they have no known competing financial interests or personal relationships that could have appeared to influence the work reported in this paper.

## Appendix A Supplementary data

Supplementary Table 1

Supplementary Table 1 Note
