# Supplementary material for: Lithium as a disease-modifying therapy for Parkinson’s disease: mechanisms, preclinical evidence, and clinical prospects
Source: Clin Park Relat Disord. 2026 Apr 9;14:100440. doi: 10.1016/j.prdoa.2026.100440 (PMC13187593; doi:10.1016/j.prdoa.2026.100440)
Supplement: Supplementary Table 1 Note [file mmc2.docx]

**Note:** 3-MA; 3-methyladenine. 3-NT; 3-nitrotyrosine. 6-OHDA; 6-hydroxydopamine. Aβ; amyloid-beta. AChE; acetylcholinesterase. AD; Alzheimer's disease. ADC; apparent diffusion coefficient. Akt; protein kinase B. ALS; amyotrophic lateral sclerosis. APP; amyloid precursor protein. BBB; Basso, Beattie, and Bresnahan locomotor rating scale. Bcl-2; B-cell lymphoma 2. BD; bipolar disorder. BDNF; brain-derived neurotrophic factor. βArr2; beta-arrestin 2. CHS; cholinesterase. CON; control. Cdk5; cyclin-dependent kinase 5. CGCs; cerebellar granule cells. CGNs; cerebellar granule neurons. DA; dopamine. DNMT1; DNA methyltransferase 1. DTI; diffusion tensor imaging. EAE; experimental autoimmune encephalomyelitis. EC50; half maximal effective concentration. FA; fractional anisotropy. FOXO3a; forkhead box O3a. FXS; fragile X syndrome. GRP78; glucose-regulated protein 78. GSH; glutathione. GSH-Px; glutathione peroxidase. GSK-3α; glycogen synthase kinase-3 alpha. GSK-3β; glycogen synthase kinase-3 beta. H₂O₂; hydrogen peroxide. HD; Huntington's disease. HFD; high-fat diet. HNE; 4-hydroxynonenal. HSP70; heat shock protein 70. IL; interleukin. IMPase; inositol monophosphatase. Ins(1,4,5)P3; inositol 1,4,5-trisphosphate. IP3; inositol trisphosphate. IPPase; inositol polyphosphate 1-phosphatase. i.p.; intraperitoneal. JNK; c-Jun N-terminal kinase. Ki; inhibition constant. LC3; microtubule-associated protein 1A/1B-light chain 3. LC3-II/I; LC3-phosphatidylethanolamine conjugate to LC3-I ratio. LiCl; lithium chloride. MB; methylene blue. MCI; mild cognitive impairment. MDA; malondialdehyde. miR; microRNA. MPP+; 1-methyl-4-phenylpyridinium. MPTP; 1-methyl-4-phenyl-1,2,3,6-tetrahydropyridine. MS; multiple sclerosis. mTOR; mammalian target of rapamycin. MWM; Morris water maze. NDD; neurodegenerative disease. NF-κB; nuclear factor kappa B. NMDA; N-methyl-D-aspartate. NSC; neural stem cells. OFT; open field test. ORT; object recognition test. p38; p38 mitogen-activated protein kinase. PD; Parkinson's disease. PEI; polyethylenimine. PFC; prefrontal cortex. PI3K; phosphoinositide 3-kinase. PKA; protein kinase A. PKC; protein kinase C. PP2A; protein phosphatase 2A. PPARγ; peroxisome proliferator-activated receptor gamma. PQ; paraquat. p-tau; phosphorylated tau. ROS; reactive oxygen species. s.c.; subcutaneous. SCA1; spinocerebellar ataxia type 1. SCI; spinal cord injury. SMD; standardized mean difference. SNCA; alpha-synuclein gene. SNpc; substantia nigra pars compacta. SOD; superoxide dismutase. SOD1; superoxide dismutase 1. TGF-β1; transforming growth factor beta 1. TH; tyrosine hydroxylase. TNF-α; tumor necrosis factor alpha. VEGF; vascular endothelial growth factor. WNT; wingless-related integration site.
